# Serum Levels of Asymmetric Dimethylarginine, Vascular Endothelial Growth Factor, and Nitric Oxide Metabolite Levels in Preeclampsia Patients

**DOI:** 10.1155/2013/104213

**Published:** 2013-09-11

**Authors:** Marjan Noorbakhsh, Maryam Kianpour, Mehdi Nematbakhsh

**Affiliations:** ^1^Water & Electrolytes Research Center, Isfahan University of Medical Sciences, Isfahan 81745, Iran; ^2^Department of Obstetrics and Gynecology, Isfahan University of Medical Sciences, Isfahan 81745, Iran; ^3^Nursing & Midwifery Care Research Center, Isfahan University of Medical Sciences, Isfahan 81745, Iran; ^4^Department of Midwifery, Isfahan University of Medical Sciences, Isfahan 81745, Iran; ^5^Department of Physiology, Isfahan University of Medical Sciences, Isfahan 81745, Iran; ^6^Isfahan-MN Institute of Basic & Applied Sciences Research, Isfahan 81546, Iran

## Abstract

*Background*. Hypertensive disorder generally complicates 5–10 percent of all pregnancies. Angiogenic growth factors may be helpful for the diagnosis and prediction of preeclampsia. Therefore, in this study we attempted to determine the serum levels of asymmetric dimethylarginine (ADMA), vascular endothelial growth factor (VEGF), and nitric oxide (NO) metabolite (nitrite) in preeclampsia patients and compared the levels with those obtained from normal pregnant women. *Methods*. Ninety pregnant women (19–33 years old) in two groups of preeclampsia and normal were considered during 2012. The levels of ADMA, VEGF, and nitrite were measured in maternal serum samples using ELISA kits. *Results*. Significant increase of VEGF and nitrite levels was observed in preeclampsia patients when compared with other groups (*P* < 0.05). The serum level of ADMA demonstrated a similar increased trend in preeclampsia patients; however, the increase was not statistically significant (*P* = 0.08). *Conclusion*. The findings reveal that the elevation of serum levels of VEGF and nitrite and possibly ADMA may be involved in the pathogenesis of preeclampsia.

## 1. Introduction

Hypertensive disorder is generally the most common disease and cause of death in pregnancy and complicates 5–10% of all pregnancies. Furthermore, the pregnancy syndrome preeclampsia complicates approximately 3–7% of all pregnancies, and the syndrome becomes a major contributor of death [1–4]. Preeclampsia generally is recognized by deficient uteroplacental perfusion [5, 6], and some risk factors such as obesity, multifetal gestations, maternal age above 35, and African-American ethnicity are associated with this syndrome [7–9]. The association between pregnancy and hypertension and its effects on mother and child life remain a subject of intensive researches for many years, and no definite solution was achieved yet. Preeclampsia often affects young and nulliparous women, and its incidence is influenced by race and genetic predispositions [10]. The role of the renin-angiotensin system and angiogenic and antiangiogenic biomarkers of placental origin has been described in pathogenesis of preeclampsia [11]. However, there is no definite biomarker to be considered as a sensitive parameter to control the disease severity. Nitric oxide (NO) is a potent vasodilator that is synthesized from L-arginine by the NO synthase and endothelial cells [12] and regulates the vascular tone and blood flow in the vascular smooth muscles. The endothelium-released NO is influenced by hypertension, and it is considered as a factor of endothelial functions [13, 14]. NO could promote the release of an important angiogenesis mediator; vascular endothelial growth factor (VEGF). VEGF is a potent endothelial cell-specific mitogen that promotes vascular hyperpermeability, and vasodilation [15–18]. Asymmetric dimethylarginine (ADMA) is an antiangiogenesis factor [19, 20] that decreases the expression of VEGF in endothelial cells [21] and may interrupt the NO-producing activity of NO synthase. 

Chedraui et al. measured the serum levels of NO, ADMA, and VEGF in severe preeclampsia and found positive correlation between NO and ADMA among preeclampsia cases, and the NO levels were significantly different in the artery and vein of umbilical vessels. Moreover, the level of VEGF was significantly lower in the artery but not in the vein in preeclampsia cases [22]. Other studies indicated an increase in ADMA level and a decrease in NO levels [23] or decrease in the level of both factors [24] in preeclampsia. Furthermore, the importance of NO has been emphasized in preeclampsia [25, 26]. ADMA may have a clinical significance [27, 28], and it is a potential biomarker to identify pregnant women at the risk of developing preeclampsia [29]. Angiogenic growth factors such as VEGF may be helpful for the diagnosis and prediction of preeclampsia [30], and it may be responsible for impaired vascular development [31]. Still, there is no well-documented data that explains how NO, ADMA, and VEGF levels vary in preeclampsia patients. So, we attempted to determine the serum levels of these biomarkers in serum samples of preeclampsia patients and compared them with those obtained from normal pregnant women. 

## 2. Materials and Methods

### 2.1. Patients

This study included patients with and without preeclampsia referred to obstetrics and gynecology clinics of Isfahan University of Medical Sciences during 2012. This study was approved by the Ethical Committee of Isfahan University of Medical Sciences.

All pregnant subjects were nulliparous healthy women without any known preexisting medical complications. Exclusion criteria included multiple gestation, prior preeclampsia, and preexisting medical conditions such as diabetes, chronic hypertension, and renal disease. Forty-five women with preeclampsia (aged 24.7 ± 0.6 years) and 45 women without preeclampsia (aged 24.5 ± 0.5 years) were selected. Preeclampsia was diagnosed by the presence of gestational hypertension, proteinuria, and hyperuricemia beginning after the 20th week of pregnancy with resolution of gestational hypertension and postpartum proteinuria. Gestational hypertension was defined as a new onset increase in blood pressure including an absolute systolic blood pressure (BP) ≥140 mmHg and/or diastolic BP ≥ 90 mmHg after 20 weeks of gestation. Proteinuria was defined as ≥300 mg per 24-hour urine collection, ≥2 + protein on voided urine sample, ≥1 + protein on catheterized urine specimen, or a protein-creatinine ratio of ≥0.3. The official informed consent was obtained from all subjects, and the demographic data were collected. 

### 2.2. Measurements

Maternal blood sample was obtained and centrifuged. The serum samples were collected and stored at −20°C for later analysis. The serum levels of VEGF were measured using an enzyme immunoassay kit (Immuno-Biological Laboratory Co., Japan). Briefly, the kit is a solid-phase sandwich ELISA using specific polyclonal and monoclonal antibodies, and the coloring agent was tetramethylbenzidine (TMB). The absorbance at 450 nm is in proportion to the VEGF concentration using the standard curve. 

The serum levels of nitrite (stable NO metabolite) were measured using a colorimetric assay kit (Promega Corporation, USA) that involves the Griess reaction. Briefly, after adding sulphanilamide solution and incubation, *N*-1-naphtylethylenediamine dihydrochloride solution was added. Then, the absorbance was measured by a microreader at 540 nm wavelength. The nitrite concentrations of the samples were determined by comparison to the nitrite standard reference curve. 

The serum levels of ADMA were measured using an enzyme immunoassay kit (DLD Diagnostika GmbH, Germany). Briefly, ADMA in the samples competes with solid phase-bound ADMA for a fixed number of rabbit anti-ADMA. The anti-rabbit/peroxidase was used to detect the antibody bound to the solid-phase ADMA, which is inversely proportional to the ADMA concentration of serum or peritoneal fluid.

### 2.3. Statistical Analyses

The data are reported as mean ± SEM. The parameters between case and control groups were compared using unpaired *t*-tests, and *P* value less than 0.05 was considered statistically significant.

## 3. Results

**Figure 1:**
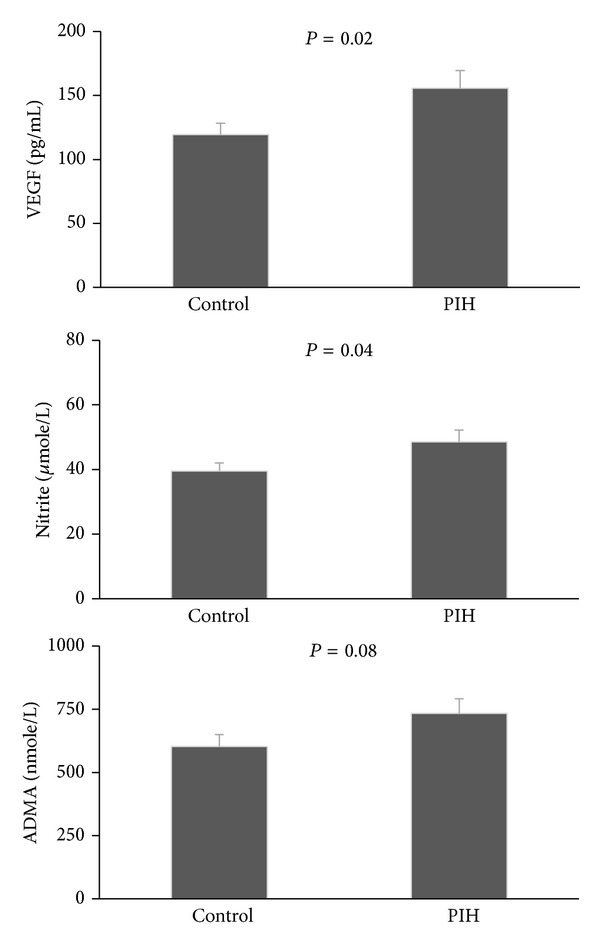
Serum levels of VEGF, nitrite, and ADMA in patients with PIH with normal matched pregnant women. The data is shown as mean ± SEM and was compared using two-tailed Student's *t*-test analysis.

The data of serum levels of VEGF, nitrite, and ADMA for the patients with preeclampsia (case) and pregnant women without preeclampsia (control) is demonstrated in Figure 1. A significant increase in VEGF and nitrite levels was observed in preeclampsia patients when compared with the control group (*P* < 0.05). The serum level of ADMA demonstrated a similar increased trend in preeclampsia patients, but the difference was not statistically significant (*P* = 0.08).

## 4. Discussion

Our observation indicated that serum levels of VEGF and nitrite were higher in patients with preeclampsia (case) than normotensive pregnant women (control). In addition, the serum level of ADMA was nonsignificantly different between the case and control groups. The increase of VEGF in preeclampsia patients was reported before [32, 33] due to endothelial activation. However, the level of free VEGF decreased in preeclampsia subjects possibly because of endothelial dysfunction [34]. In the current study, we measured the total VEGF; bounding and free levels in serum, which was detectable by ELISA assay. Therefore, higher levels of VEGF in preeclampsia subject were expected which is consistent with the findings reported in previous studies [32, 33]. Moreover, NO level increases in hypertension condition, and about 50% of our patients had a blood pressure above 160/90 mmHg, which may increase the serum level of NO. However, there are some controversies for alteration of NO in preeclampsia patients. Some reports indicated decreased NO level [35–39], and no change in NO level was detected by others [40–44]. The reason for such observation may be related to increase of nitrite excretion in the urine or decrease of placental NO synthase activation [45] in pregnant women with preeclampsia. In addition, it is also reported that the serum level of NO is related to gestational age, which become at maximum level in gestational age of 35 to 36 weeks [46]. We measured the serum level of nitrite as one of the stable NO metabolite, which was increased in the case group. Two reasons may contribute to such finding. First, the gestational age of our patients was 34.8 ± 2.4 weeks, and possibly at this age the serum level of NO was high [46], and second, we measured one of the NO metabolites, nitrite. Different results would possibly be obtained if other metabolite; nitrate was also measured. One limitation for our study was lack of gestational age matching between the two groups because the preeclampsia patients were included in the study as soon as the sign of disease appeared. 

ADMA is an antiangiogenesis factor that decreases VEGF expression in endothelial cells and prevents the formation of NO from NOS. Increase of ADMA in endothelium-dependent vascular dysfunction was detected in diabetic, hypertensive, and hypercholesterolemic patients [20, 47, 48]. It is reported that ADMA may decrease at the beginning of normal pregnancy [49], but under the preeclampsia condition its concentration increases [50–52], which may be associated with the disease intensity [50]. It seems that ADMA is involved in preeclampsia pathogenesis because inhibition of NO synthesis in rodents during pregnancy produces signs similar to those of preeclampsia [53–55]. In the current study, the serum level of ADMA nonsignificantly increased in the case group. The reason for such nonsignificant elevation may related to the increase of NO level in these patients. 

## 5. Conclusion

The findings reveal that elevation of serum levels of VEGF and nitrite and possibly ADMA may be involved in the pathogenesis of preeclampsia, and these markers may be helpful for the diagnosis of the disease.
